# Editorial: Prevention and treatment of metabolic diseases using bioactive metabolites of herbal medicines also used as foods

**DOI:** 10.3389/fphar.2026.1779704

**Published:** 2026-02-02

**Authors:** Rong-Rui Wei, Wen-Min Liu, Chun-Su Yuan, Chun-Lei Zhang, Zhi-Pei Sang, Qin-Ge Ma

**Affiliations:** 1 College of Medicine and Health Science and School of Life Science and Technology, Wuhan Polytechnic University, Wuhan, China; 2 College of Chemistry and Pharmaceutical Engineering, Nanyang Normal University, Nanyang, China; 3 Department of Anesthesia and Critical Care, Tang Center for Herbal Medicine Research, Committee On Clinical Pharmacology and Pharmacogenomics, Pritzker School of Medicine, The University of Chicago, Chicago, IL, United States; 4 School of Traditional Chinese Pharmacy, China Pharmaceutical University, Nanjing, China; 5 School of Pharmaceutical Sciences, Hainan University, Haikou, China

**Keywords:** hepatoprotection, liver disease, mechanism, pathogenesis, target, treatment

Liver disease is a general term for lesions that occur in the liver. Its pathogenesis is complex and mainly related to factors such as viral infection, metabolic abnormalities, drug effects, and alcohol intake (Zhong et al., 2025). These factors can lead to liver cell damage, inflammatory response, and fibrosis process (Bai et al., 2025). The main classifications of liver diseases include NAFLD, drug-induced liver injury, viral hepatitis, and liver cancer (Ma et al., 2025). The liver disease is extremely harmful, with hidden early symptoms and the possibility of developing into cirrhosis, liver failure, and even liver cancer in the later stages. This disease seriously endangers human health (Xiao et al., 2025). The current treatment of liver disease has made certain progress, and the combination of immunotherapy and targeted therapy for liver cancer significantly prolongs the survival of patients. The approval of the first MASH targeted drug marks a new stage in the treatment of metabolic liver disease, but there are still limitations (Ali and Lai, 2025). Antiviral drugs used to treat liver disease have a risk of drug resistance and a high recurrence rate after discontinuation. Some drugs used to treat liver diseases have high prices, drug resistance risks, and side effects (McNally and Carey, 2025). However, traditional Chinese medicine with the same origin of medicine and food has shown unique advantages in the treatment of liver disease (Ma et al., 2023). These natural medicines can regulate liver metabolism and reduce inflammatory reactions through multiple targets. It also has minimal side effects and is suitable for long-term regulation. It can effectively assist in improving liver function (Ma et al., 2024). In the future, the treatment of liver diseases will develop towards precision, intelligence, and minimally invasive methods (Ghosh et al., 2025). Gene editing, regenerative medicine, and other technologies are expected to break through existing treatment bottlenecks (Nair et al., 2025). Digitized liver resection technology and multidisciplinary integrated diagnosis and treatment models will further enhance treatment effectiveness, bringing new hope to liver disease patients.

Xu et al. evaluated the effects of rhein on inflammation, renal function, and gut microbiota induced by high-fat diet in C57BL/6J mice. The mice were fed a 60% fat diet for 12 weeks to establish an obesity related glomerular model. After continuous feeding of rhein (300 mg/kg/day) for 12 weeks, serum cytokines (IL-6, TNF-α), renal tissue pathology, and 16S rRNA microbiome sequencing were measured and analyzed. The research results indicated that rhein significantly reduced body weight, serum triglycerides, proteinuria, and improved glomerular lesions in mice, and it also significantly reduced the levels of serum IL-6, TNF-α, and creatinine. It was found that rhein restored the diversity of gut microbiota (Chao1 index increased from 303.58 to 425.78) and reversed the imbalance between Firmicutes and Bacteroidetes through 16S rRNA sequencing. The study found that significant differences in microbial community structure (between the rhein group and the model group) were confirmed through similarity analysis (R = 0.926, p = 0.008). In a word, rhein occasionally alleviated the progression of obesity-related glomerulopathy, and confirmed that this was related to factors such as anti-inflammatory, lipid-lowering, and microbial community regulation mechanisms.

Li et al. used LC-MS, computer simulation docking analysis, and molecular dynamics to study the inhibition of cytochrome P450 enzymes by triterpenoids of *Ganoderma lucidum*, and evaluated their interference with clinical drug metabolism processes. It was found that triterpenoids of *G. lucidum* inhibited multiple CYP450 subtypes and interfered with the pharmacokinetics of some drugs in rats. This study also evaluated the inhibitory effects of triterpenoids on CYP 1A2, 2D6, 3A4, 2A6, 2B6, 2C9, and 2C19. The results showed that lanostane triterpenoids significantly inhibited CYP 1A2, indicating a strong affinity for these compounds. It was also found that some triterpenoids of *G. lucidum* only showed extensive inhibitory effects on CYPs, except for CYP 3A4, 2D6, 2C9, and 2C19. In addition, LC-MS, computer simulation docking analysis, and molecular dynamics studies displayed that some triterpenoids of *G. lucidum* interfered with clinical metabolism by inhibiting CYPs *in vitro*, which probably induced potential drug interactions. The above research provides important reference value for the promotion of human health by triterpenoids of *G. lucidum*.

Miao et al. used a high-fat diet (HFD, 60% kcal fat) to establish obesity in C57BL/6J mice and investigate the effect of *Portulaca oleracea* L. extract on the TLR4/NF-κB signaling pathway. This study used intestinal tissue from mice to detect relevant indicators, and analyzed protein expression through Western blotting and immunohistochemistry. ELISA kits were used to detect pro-inflammatory cytokines. In this study, UPLC and ESI-Q-TOF-MS were used to detect the chemical components of *P. oleracea* L. extract. It was found that chronic oral administration of *P. oleracea* L. extract ameliorated colon shortening syndrome and reduced bowel inflammation in HFD-induced obese mice by suppression of TLR4 and NF-κB signaling pathway to downregulate TLR4, MyD88, Ser32 phosphorylation of NF-κB inhibitor alpha, and Ser536 phosphorylation of NF-κB p65 expression levels, thereby inhibiting the pro-inflammatory cytokines, TNF-α, and IL-6 levels. This study found that *P. oleracea* L. extract inhibited the TLR4/NF-κB signaling pathway by regulating obesity induced intestinal inflammation, which provided significant research value for *P. oleracea* L. extract against intestinal-inflammation-associated obesity.

Li et al. made a review on natural compounds in the treatment of polycystic ovary syndrome (PCOS). PCOS is a multifaceted endocrine disorder that is typically associated with human metabolism (such as insulin resistance). To some extent, this disease increases the risk of type 2 diabetes, diabetes nephropathy, endometrial cancer, cardiovascular disease, and dyslipidemia. So far, there is no standardized treatment for PCOS. The existing therapies mainly rely on personalized drug therapy and lifestyle modifications. However, these treatments often lead to adverse reactions or ineffective results. However, natural compounds are highly valued by researchers due to their hormone regulating effects and low toxicity. It is found that some natural compounds, such as inositol, resveratrol, berberine, and quercetin, alleviated symptoms of PCOS. Their multi-target nature overcomes the limitations of single target drugs and has potential research value. This review comprehensively analyzed the application and intervention of natural compounds in the treatment of PCOS, and evaluated the impact of natural compounds on the prevalence and therapeutic potential of PCOS.

Hu et al. validated the anti-functional dyspepsia effects of Citri Reticulatae Pericarpium through network analysis and experimental verification. Functional dyspepsia is a common functional gastrointestinal disease, and there is currently no specific medication for its treatment. Although Citri Reticulatae Pericarpium is a commonly used traditional Chinese medicine for relieving bloating and spleen stomach disharmony, its efficacy and molecular mechanism in treating functional dyspepsia still need further research. This study validated the potential mechanism of Citri Reticulatae Pericarpium in combating functional dyspepsia through HPLC, ESI-Q-TOF-MS, network analysis, and experiments. It was found that 90 metabolites of Citri Reticulatae Pericarpium were identified by HPLC-ESI-Q-TOF-MS and 70 targets interacted with functional dyspepsia were found in this study. This *in vivo* experiment showed that Citri Reticulatae Pericarpium effectively improved body weight, gastric emptying rate, intestinal transport rate, and pathological gastric tissue structure, and downregulated IL-6, TNF-α, and IL-1*β* levels. In addition, Citri Reticulatae Pericarpium increased the abundance of Patescibacteria, *Bacteroides*, and Proteobacteria. In summary, Citri Reticulatae Pericarpium combated functional dyspepsia by reducing inflammation, activating TLR4/MyD88 and MAPK signaling pathways, and regulating gut microbiota structure and composition.

Lai et al. studied that Thonningianin A alleviated APAP induced hepatotoxicity by activating GPX4 and modulating endoplasmic reticulum stress. As is well known, excessive use of APAP can lead to acute liver injury. In order to find safer and more effective ways to treat acute liver injury, this study established an acute liver injury model in C57BL/6 mice by administering doses of 20 mg/kg and 40 mg/kg of APAP, respectively, to verify that Thonninginin A alleviated APAP induced liver toxicity. It was found that Thonninginin A significantly reduced ALT and AST levels, inhibited the production of inflammatory cytokines, and reduced stress markers in oxidized liver tissue. In addition, Thonningianin A reduced CHOP and BAX levels by increasing BCL-2 expression, mainly by downregulating endoplasmic reticulum to alleviate endoplasmic reticulum stress GRP78, p-PERK, and ATF4. It was worth noting that Thonninginin A achieved liver specific GPX4 knockout through AAV-8 mediated shRNA delivery, emphasizing the important role of GPX4 in mediating liver protection. These findings indicated that Thonninginin A was a promising hepatoprotective therapeutic agent, and its unique oxidative action had a very good effect in preventing and treating APAP induced liver injury.

Chen et al. evaluated traditional Chinese medicine formulas for COVID-19 from 9 databases through randomized controlled trials, and compared randomized controlled trials that recruited participants during and after the first wave of COVID-19. The research results revealed that the average scores of CONSORT 2010, CONSORT-CHM Formulas 2017, and checklist of sub-questions based on the CONSORT-CHM Formulas 2017 were 16.4, 15.2, and 17.2 respectively. The reporting rates for sample size calculation, allocation concealment, and blinding were all below 30%. Using the checklist of sub-questions based on the CONSORT-CHM Formulas 2017 facilitated a more accurate analysis and evaluation of CHM intervention schemes. Meanwhile, subjects recruited during the first wave of the COVID-19 pandemic were at a higher risk of participant blinding compared to studies with later recruitment (*P* < 0.05). Due to the suboptimal quality of methodological reporting in randomized controlled trials of CHM formulations for COVID-19, further improvements in sample size calculation, allocation concealment, and blinding are needed in-depth research in the future.

Cheng et al. elucidated that phenylpropanoids of *Zea mays* L. improved metabolic disorders in diabetic mice by regulating gut microbiota. The phenylpropanoids of *Z. mays* L. were identified by HPLC-QTOF-MS and NMR. Targeted serum metabolomics studies were conducted using UHPLC-LTQ Orbitrap MS in this work, and 16S rRNA analysis was employed to elucidate the mechanisms by regulating lipid and glucose metabolism. It was found that phenylpropanoids of *Z. mays* L. significantly reduced oral glucose tolerance test values, insulin, and blood glucose levels at a dose of 160 mg/kg/day (*P* < 0.001). The phenylpropanoids of *Z. mays* L. lowered the insulin resistance index, reversed the accumulation of glycogen in the liver, and reduced lipid deposition in the liver. The 16S rRNA sequencing results indicated that phenylpropanoids of *Z. mays* L. interacted with gut microbiota, exerting a positive regulatory effect on bifidobacteria, and lactobacilli. Therefore, phenylpropanoids of *Z. mays* L. have great potential as adjuvant therapy for complex metabolic diseases such as diabetes.

Leonardo et al. found that Delites™ prevented metabolic syndrome in male SD rats induced by high cholesterol and high-fat diet, and regulated their gut microbiota in a randomized preclinical trial. Delites™ is a supplement closely related to traditional Chinese medicine, which holds potential development value in regulating intestinal microbiota and alleviating metabolic syndrome. In this study, SD rats were divided into 4 groups: control-normal, CFED, CFED + low-dose Delites™, and CFED + high-dose Delites™. Through analysis of lipid profile, enzyme activity, molecular biomarkers, and gut microbiota, it was found that Delites™ significantly improved the lipid profile, reduced TNF-α level, enhanced IL-10 expression, and increased PGC-1*α* level. Meanwhile, gut microbiota regulation showed an increase in bifidobacteria and lactobacilli, accompanied by a decrease in *proteus*. These studies have found that Delites™ has potential in precision medicine to combat metabolic disorders, and further research is needed to investigate its long-term effects and translational relevance for humans in the future.

Xu et al. studied the therapeutic effect of “Gui-Zhi-Ge-Gen-Tang” on T2DM based on pharmacodynamics and pharmacokinetics. In this study, the decoction of “Gui-Zhi-Ge-Gen-Tang” was prepared using water extraction, and its chemical components were analyzed using UPLC-Q-TOF-MS and HPLC. This study established a T2DM model by intraperitoneal injection of streptozotocin, administered drugs by gavage for 6 consecutive weeks, and used ELISA to detect serum markers. In this study, the abundance of gut microbiota in rats was detected through 16S rDNA sequencing, the content of short-chain fatty acids in rat feces was measured using GC-MS, and the expression of GPR43 and GLP-1 proteins in rat colon tissue was assessed using Western Blot. Meanwhile, this study established a Caco-2/HT29 cell model to measure the TEER and ALP activity to evaluate the integrity and polarization of the cell model, and investigated the bidirectional transport mechanism of “Gui-Zhi-Ge-Gen-Tang” decoction. The research results indicated that the decoction of “Gui-Zhi-Ge-Gen-Tang” stimulated the intestinal flora, increased the content of short-chain fatty acids, activated GPR43 protein, and promoted the secretion of GLP-1 in intestinal L cells. This study demonstrated the scientificity and rationality of treating T2DM with the decoction of “Gui-Zhi-Ge-Gen-Tang” from the perspectives of pharmacodynamics and pharmacokinetics.

Guohui et al. revealed the mechanism of treating hyperlipidemia with *Fagopyrum tataricum* and kiwi juice co-fermentation products by utilizing transcriptomics and metabolomics. In this study, *F. tataricum* was co-fermented with kiwi juice, and the flavonoids in the fermentation product were analyzed through non-targeted metabolomics. Transcriptomics and metabolomics were utilized to investigate the anti-hyperlipidemic effect of the fermentation product in zebrafish fed a high-fat diet. The research results revealed that the co-fermentation product was rich in quercetin, luteolin, rutin, and kaempferol, which significantly reduced lipid accumulation in zebrafish. Metabolomics revealed 24 core differentially expressed metabolites, including glycerophospholipids, sphingolipids, glycerides, and fatty acyls. Transcriptomic analysis showed that the co-fermentation product regulated genes such as PLTP, ApoC1, SOAT2, SCARB1, PLA2G12B, and HMGCRa, and these genes were related to cholesterol metabolism and pathways associated with fat digestion and absorption. Therefore, this study indicated that the co-fermentation product of *F. tataricum* and kiwi juice enhanced the bioavailability of flavonoids and held great potential in preventing and treating hyperlipidemia.

Wu et al. assayed that hydroxysafflor yellow A alleviated oxidative stress and inflammatory damage in the mice with NAFLD, and regulated their gut microbiota. In this study, the liver function and lipid metabolism were assessed by measuring ALT, AST, TC, and TG levels in NAFLD mice induced by HFD. The levels of IL-6, TNF-*α*, and IL-1*β* in serum were determined by ELISA. The expression of the NLRP3 inflammasome and its downstream effector Caspase-1 in the liver was analyzed by Western blotting. Liver tissue was subjected to histopathological examination using H&E staining to assess structural damage. In addition, 16S rDNA sequencing and non-targeted metabolomics analysis of fecal samples were conducted to elucidate the enriched metabolic pathways associated with hydroxysafflor yellow A treatment. The research findings revealed that hydroxysafflor yellow A significantly inhibited HFD-induced weight gain, alleviated liver inflammation, and reduced ALT, AST, TG levels (*P* < 0.05). It also decreased the mRNA and protein expression of NLRP3, caspase-1, and IL-1*β* in the liver, and increased SOD activity (*P* < 0.05). Analysis of gut microbiota revealed a significant increase in the abundance of Turicibacter and a decrease in the abundance of Ruminococcus. Therefore, hydroxysafflor yellow A exhibited potent anti-inflammatory and anti-oxidant effects, effectively alleviating liver inflammation and oxidative stress in NAFLD mice. Its therapeutic mechanism may be related to regulating intestinal flora and modulating serum phenylalanine and tyrosine metabolism.

Cao et al. conducted a review on the effects of traditional Chinese medicine preparations on patients with T2DM complicated fatty liver. The Controlled Attenuation Parameter (CAP), as an important means of evaluating steatosis in MAFLD, can also be used to assess metabolism-related fat parameters. In this review analysis, researchers consulted relevant databases to obtain clinical randomized controlled trial data on the treatment of T2DM with MAFLD using traditional Chinese medicine preparations. Data analysis was conducted using RevMan 5.4 software, and the results were presented in the form of forest plots. A total of 599 papers were retrieved in this study, and 8 trials (involving 648 participants) were selected for inclusion. The analysis revealed that the traditional Chinese medicine preparation significantly reduced the CAP value in patients with T2DM complicated by MAFLD (MD = −15.19, CI [−22.53, −7.85], *P* < 0.0001). Therefore, traditional Chinese medicine preparation effectively reduced the CAP value of T2DM combined with MAFLD, demonstrating significant therapeutic effects on glucose and lipid metabolism as well as liver function. This indicated that traditional Chinese medicine preparation reduced hepatic fat deposition, restored hepatocyte function, and exhibited a favorable therapeutic effect on T2DM combined with MAFLD.

Cesar et al. found that citrus flavonoids enhanced the control of blood glucose and metabolism in patients with prediabetes treated with metformin. In a 12-week randomized and double-blind trial, participants received treatment with metformin combined with a citrus flavonoid supplement (250 mg/day). At the end of the intervention, it was found that the nutritional supplement group was able to improve postprandial glucose metabolism, reduce OGTT levels, and maintain GLP-1 levels. In contrast, the placebo group showed a decrease in GLP-1 level and an increase in insulin resistance. This flavonoid supplement reduced TNF-*α* level, enhanced plasma antioxidant capacity, and decreased indicators such as body weight, fat mass, and BMI (*P* ≤ 0.05). In summary, citrus flavonoids can improve postprandial blood glucose level, maintain GLP-1 level, reduce inflammation and oxidative stress, and improve blood pressure level, thus possessing potential research value in the treatment of early-stage T2DM.
